# Barriers and Facilitators to Older Adults’ Engagement in Social Prescribing: A Qualitative Study Using Focus Groups

**DOI:** 10.3390/jcm14134780

**Published:** 2025-07-07

**Authors:** Rute Sadio, Adriana Henriques, Paulo Nogueira, Andreia Costa

**Affiliations:** 1 Nursing Research, Innovation and Development Centre of Lisbon (CIDNUR), Nursing School of Lisbon (ESEL), 1600-190 Lisbon, Portugal; ahenriques@esel.pt (A.H.); paulo.nogueira@esel.pt (P.N.); andreia.costa@esel.pt (A.C.); 2 ULSAC—Unidade Local de Saúde do Alentejo Central, UCSP Estremoz, 7000-811 Évora, Portugal; 3 Instituto de Saúde Ambiental (ISAMB), Faculty of Medicine, University of Lisbon, 1649-028 Lisbon, Portugal; 4 Laboratório para a Sustentabilidade do Uso da Terra e dos Serviços dos Ecossistemas—TERRA, 1349-017 Lisbon, Portugal

**Keywords:** social prescribing, older adults, COM-B model, barriers and facilitators, community engagement, qualitative research

## Abstract

**Introduction**: Social prescribing is an innovative approach that connects individuals to community-based activities to promote well-being. This study explores the barriers and facilitators influencing older adults’ engagement in social prescribing programmes in Portugal. **Methodology**: A qualitative study was carried out in October 2024, in Portugal, with 16 participants aged 65 and over. Data was collected through two focus groups, each with eight participants. Data were analysed using the COM-B model (Capability, Opportunity, Motivation) to identify key factors affecting adherence. **Results**: The main barriers identified were physical limitations, digital exclusion, transport inaccessibility, and the urban-centric location of services. Facilitators comprised tailored activities, digital support and education, accessible venues and transport, and personalised interventions. Ongoing feedback mechanisms and familiar community settings were essential for sustained participation. **Conclusions**: These findings suggest that co-designed, inclusive, and locally accessible programmes can significantly enhance the involvement and well-being of older adults.

## 1. Background

The limitations of the traditionally dominant biomedical model have become increasingly evident, particularly with regard to its inability to address the psychological, social and environmental determinants of health [1,2]. As a result, non-pharmacological and holistic interventions have gained prominence in addressing emotional, social, spiritual, and contextual dimensions of well-being [3,4].

In response to the multifaceted needs of individuals, complex interventions have emerged. These are defined by the interplay of multiple components and their influence across individual, community-based, and systemic levels [2,5]. One such intervention is social prescribing—a strategy that connects individuals to non-clinical, community-based activities such as support groups, arts programmes, and exercise initiatives, all aimed at promoting well-being and preventing illness [2,6].

Social prescribing is an innovative model that emphasises the active participation of patients in community life, fostering holistic, person-centred care [6]. It positions individuals at the heart of care, engaging them in both the planning and delivery of interventions. It enables healthcare professionals to adopt a more integrated approach, recognising the patient’s unique circumstances and preferences, in contrast to the fragmented focus of traditional biomedical models [7,8].

A central feature of the person-centred care is the active involvement of patients in decisions concerning their health. This engagement fosters greater health literacy and self-management capacity, empowering individuals to make informed choices. When patients are involved in decision-making, they report higher confidence and motivation to adhere to recommendations and engage in activities that enhance their well-being [9,10].

As a person-centred strategy, social prescribing emphasises the co-creation of care, viewing the individual as an active partner in their health journey. This intails listening to concerns, respecting preferences, and jointly determining the course of action [11]. Such engagement empowers the patient and associates with improved health outcomes [12].

The outcomes of social prescribing span multiple domains, including physical, emotional, and psychological well-being. For older adults, participation in community-based activities—such as cultural programmes, volunteering, gardening, walking groups, and the visual arts—can enhance quality of life, self-esteem, confidence, and resilience. These initiatives also mitigate loneliness, support mental health, and may contribute to the prevention of cognitive decline [6,13].

Social prescription is also a mechanism for behaviour change, aligning with the COM-B model [14], which posits that Capability, Opportunity, and Motivation are necessary conditions for the adoption of new behaviours. These interventions promote healthier lifestyles, enhance self-care, increase motivation, and improve health literacy—particularly among older populations [15,16]. They also contribute to reduced dependency on medication and alleviate pressure on primary care services, resulting in cost savings and system-wide efficiencies [6,9,11]. The COM-B model thus offers a useful theoretical lens for understanding how social prescribing fosters sustainable change and generates social and economic benefits. The COM-B model (Capacity, Opportunity, Motivation-Behaviour), proposed by [14], describes three fundamental conditions for behaviour change: the psychological and physical capacity to act, the social and contextual opportunities that enable it, and the motivation to carry out such behaviour. Its flexible structure makes it possible to understand how internal and external factors interact in the adoption of new practices and is especially useful in public health and active ageing contexts. Previous studies have applied the COM-B model to understand social prescribing interventions, both from the perspective of training health professionals [17] and identifying barriers and facilitators to the involvement of people with chronic conditions, including older adults [13]. This research reinforces the model’s usefulness in analysing the behavioural and structural components that influence the success of these community interventions. The applicability of this model in ageing contexts is reinforced by its ability to incorporate physical, emotional and social factors, all of which are particularly relevant in this population.

Implementing social prescribing models facilitates collaboration between healthcare, social services, and community organisations, fostering integrated care that extends beyond the biomedical model [2]. This cross-sector synergy strengthens local community infrastructure, builds resilience, and enhances awareness of accessible resources. It also supports networks of voluntary, community, and social enterprise organisations, encouraging volunteerism and helping to meet the unmet social and emotional needs of service users [15].

Finally, social prescribing also addresses the social determinants of health, contributing to reduced isolation, enhanced individual skills, and support for marginalised populations [15,18,19]. In this way, it produces benefits that extend beyond the individual level, promoting broader community-level transformation.

This study was carried out in a Personalised Healthcare Unit (UCSP) located in the Alentejo region of Portugal, a markedly rural and ageing area. The institution offers primary health care and community services, catering for a mainly elderly population with varying levels of health literacy and a high risk of social isolation [20,21]. The choice of this context is justified by its symbolic representativeness in relation to other regions of the country facing similar challenges [22]. Understanding local dynamics allows us to infer relevant implications for the formulation of public policies and the adaptation of social prescription models to territories with similar characteristics. In Portugal, the social prescription model is still in its early stages, with pilot experiments limited to a few regions. The national health system faces structural challenges—such as an ageing population, frequent use of emergency services and low health literacy—which makes the adoption of community interventions a growing need for the elderly [23].

Understanding the facilitators and barriers experienced by older adults is essential for designing effective and inclusive social prescribing interventions. Therefore, the aim of this study was to identify these factors through a qualitative exploration of older adults’ perspectives on social prescribing.

## 2. Methods

The focus group method was chosen for its capacity to elicit in-depth discussion of key themes relevant to the design of social prescription interventions, particularly the identification of barriers and facilitators to participation. This approach is well suited to capturing rich, experience-based qualitative data and is also valued for its efficiency in generating multiple perspectives within a shared social context [24]. The research team had prior experience with qualitative methods and with older adult populations. To maintain rigour, the team engaged in reflexive practices throughout the study, explicitly acknowledging and mitigating potential biases in the collection and interpretation of data.

### 2.1. Design and Participants

This qualitative study was carried out in Portugal, specifically within a Personalized Healthcare Unit in the Alentejo region, using focus group discussions to collect data. Recruiting aimed at including six and 10 participants per group, with slight over-recruitment to account for potential absences [24]. Of the 20 participants initially invited, four declined to participate due to mobility or scheduling issues. Ultimately, two focus groups were held, each with eight older adults (total *n* = 16), aged between 68 and 94, representing both genders and from rural and urban settings. The health unit where the study was carried out currently serves around 3540 users, with a high proportion of elderly people (30%). The 16 participants selected reflect the diversity of this universe in terms of gender, age, place of residence (rural and urban) and health condition (presence or absence of chronic diseases). The choice was intentionally aimed at representing distinct patterns of experience, rather than guaranteeing statistical representativeness. Theoretical saturation was reached after the second group, when no new relevant information emerged. Group composition was organised based on availability, with efforts to ensure diversity in residence and gender (Table 1).

### 2.2. Inclusion and Sampling

Inclusion criteria required participants to be aged 65 or older, cognitively intact (i.e., no diagnosis of dementia or major neurological impairments), able to ambulate independently, and not living in long-term care facilities.

### 2.3. Sampling Approach

A purposive, non-probabilistic sampling strategy was used. The two focus groups were heterogeneous in terms of gender (men and women), age, literacy level (participants with different levels of education) and health conditions (presence or absence of chronic illnesses) in order to reflect a wide range of lived experiences. Participants were selected directly by the principal investigator from among patients at the Personalized Healthcare Unit, with whom the researcher had pre-existing professional contact. Recruitment continued until data saturation was reached—defined as the point at which no new relevant information emerged [25]—which ensured depth and richness of qualitative insights. It is acknowledged that the principal investigator had previous professional contact with some participants. To mitigate possible bias, efforts were made to ensure a neutral and respectful group environment. In addition, the data analysis was peer-reviewed collaboratively by the research team to increase validity.

### 2.4. Ethics Statement

The study was approved by the Ethics Committee of ARSA (Opinion 17/CE/2022, 22 June 2022) and conducted in accordance with the Declaration of Helsinki. Participants were individually informed of the study objectives, assured of confidentiality, and provided written informed consent, including permission for audio recording. All participants also consented to the use of anonymised quotations in publications.

### 2.5. Procedure

The study adhered to the COREQ 32-item checklist for reporting qualitative research [26]. To assess eligibility and characterise the sample, data were collected on age, gender, residence, mobility, and cognitive status.

Focus groups were conducted in October 2024 with participants recruited from primary healthcare services in the Alentejo region, specifically users of a Personalized Healthcare Unit. Sessions were held in a privacy setting, lasted approximately 50 min, and were moderated by the first author (RS). Only the researcher and participants were present. Field notes were taken immediately following each session, capturing non-verbal cues such as facial expressions and emotional responses to enrich the transcription-based analysis. Despite lasting 50 min, each focus group session was carefully structured, with a clear initial introduction to the objectives of the study and the topics to be covered. The focus group methodology was chosen over individual interviews in order to capture the dynamics of social interactions and the complementarity of opinions between the participants. The script was tested in the first group, and as no adjustments were needed, that session was included in the final analysis. The absence of formal cognitive interviews is recognised as a limitation of the study.

During the discussions, topics included participants’ understanding of their involvement in social prescribing activities and their perceptions of both barriers and facilitators to engagement. The interview guide was developed based on existing studies on social prescribing among older adults in community settings.

The facilitator received formal training in qualitative methods, both from the Doctoral Nursing Program and through mentorship by experienced researchers.

### 2.6. Question Guide

A semi-structured question guide (Table 2) was developed collaboratively by the research team, based on a comprehensive literature review and in accordance with best practices in focus group methodology. The guide aimed to elicit participants’ perspectives on the barriers and facilitators to engaging in social prescribing. The script was tested in the first focus group to assess clarity and relevance. As no adjustments were deemed necessary, this session was included in the final analysis.

Prior to beginning the discussion, participants received a brief orientation on the concept of social prescribing to establish shared understanding.

The guide was piloted during the first focus group, and no substantive changes were required; thus, the data from this session were included in the final analysis [27]. The guide included opening demographic questions, followed by core questions on facilitators, barriers, and support mechanisms, and concluded with a prompt for additional feedback.

### 2.7. Data Analysis

Data analysis followed a rigorous and systematic process, consistent with established qualitative research standards [24,28]. The analytical approach comprised three stages: (1) open coding, informed by the question guide and emerging themes; (2) thematic categorisation, where similar responses were grouped; and (3) interpretation, involving in-depth thematic analysis to uncover patterns and meanings.

Focus group recordings were transcribed verbatim, and transcripts were analysed using MAXQDA (Version 24.5) software. During the initial analysis stage, all the relevant quotes derived from the focus group discussions were examined to identify recurring occurrences, with the aim of grouping them together systematically using an open coding system. Coding disagreements were resolved through discussion among researchers, ensuring consensus. All the coded data was subsequently reviewed and validated by the co-authors to ensure methodological consistency.

The analysis followed an inductive content analysis approach, allowing themes to emerge from the data rather than being pre-imposed. After the second focus group, data saturation was observed, confirming that the number of groups was sufficient to address the research question.

To ensure the trustworthiness of the findings, four quality criteria were applied [29]:

- Credibility was supported through the inclusion of a diverse participant group, detailed transcription, and careful team-based analysis.
- Transferability was enhanced via thorough descriptions of the study setting, sample characteristics, and participant experiences.
- Dependability and confirmability were established by maintaining a transparent audit trail of the methodological and analytical decisions made at each stage.

## 3. Results

**Figure 1:**
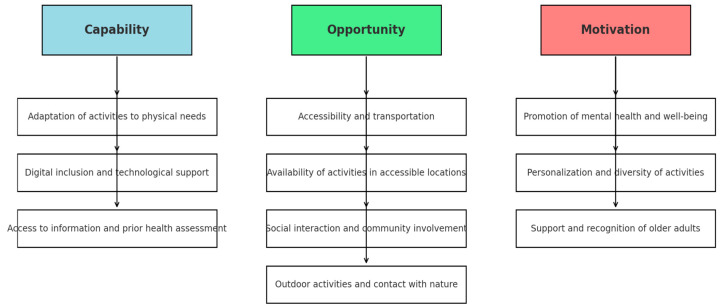
Mapping of the ten emerging themes in the domains of the COM-B model.

Analysis of the focus group data revealed nine main themes, categorised according to the COM-B model (Capability, Opportunity, Motivation). Figure 1 illustrates the relationship between the ten themes identified and the three domains of COM-B. Each sub-theme reflects a specific barrier or facilitator to the involvement of older people in social prescribing. These include adaptation of activities to physical needs, digital inclusion, access to information, transportation and accessibility, availability of venues, social interaction, contact with nature, mental health promotion, and personalisation of activities.

### 3.1. Capability

Three themes emerged under the capability domain, reflecting individual-level factors that affect older adults’ participation: adaptation to physical needs, digital inclusion, and access to information and health assessment.

#### 3.1.1. Sub-Theme 1: Adaptation of Activities to Physical Needs

Adaptation of activities to older adults’ physical capacities was a recurrent theme. Participants noted that physical challenges can limit or prevent participation, particularly when activities require high physical effort or are not adapted to their functional abilities.

Barrier: The lack of adaptation in some activities was mentioned as a significant obstacle. High-effort activities were perceived as inaccessible for older people with mobility problems, leading to exclusion.

*‘Some activities are too demanding for my state of health now—I have a lot of pain in my knees.’*

*(JPP, 75, urban)*

Facilitator: The implementation of activities adapted to the physical limitations of older adults was highlighted as a factor that supports engagement. Participants emphasised the importance of options that promote socialisation and require less physical effort.

*‘I need something that takes into account my health limitations. activities that are less intense and more geared towards being with each other.’*

*(ED, 76, urban)*

#### 3.1.2. Sub-Theme 2: Digital Inclusion and Technological Support

Digital literacy and access to technology have emerged as key factors influencing participation. Some participants described technology as a tool for maintaining social connections and reducing isolation, especially when in-person options were limited. For others, however, a lack of digital skills or access created significant obstacles.

Participants emphasised the importance of digital education and ongoing technical support as essential to ensuring equitable access to increasingly digitised community programmes.

Barrier: Difficulty in using technology was identified as an obstacle to participation. Older adults who had no prior contact with digital devices or who face motor or cognitive challenges reported that the lack of adequate support makes the use of technology even more difficult.

*‘You need to know how to use technology for many of these activities. That’s hard for some of us—I find it very difficult and honestly, I don’t like it much.’*

*(ED, 76, urban)*

Facilitator: Offering computer classes and technical support during online activities was mentioned as an effective solution for promoting digital inclusion. Many older people expressed an interest in learning to use technology better, especially when they realise that it can facilitate social contact and access to useful information and services.

*‘I’d like to see activities with technology—Something to help me to use my mobile phone better. I have a lot of doubts… computer classes would be helpful.’*

*(JP, 68, urban)*

#### 3.1.3. Sub-Theme 3: Access to Information and Prior Health Assessment

Access to clear information and individualised health assessments were also considered essential for engagement. Participants reported that not knowing about the benefits of social prescribing—or how activities could be tailored to their needs—often created uncertainty and reluctance to participate. Participants expressed a desire for more proactive communication from healthcare providers and personalised assessments to guide recommendations.

Barrier: The lack of accessible information about available activities was highlighted as a significant obstacle. Many older people reported not knowing where to look for reliable information about the options available, which makes decision-making more difficult and reduces the likelihood of participation, especially for users living in parishes.

*‘The problem is the lack of information. We don’t know where to find out what activities exist or where they’re held. I have a lot of doubts.’*

*(NM, 71, urban)*

Facilitator: Holding information sessions on the benefits of social prescribing and implementing personalised health assessments were mentioned as strategies that help adapt recommendations to the needs of the elderly. The possibility of understanding the positive impacts of these activities on health and well-being was mentioned as a factor that encourages adherence and reduces initial resistance.

*‘It would help if doctors or nurses explained the benefits of the activities—and how they can be adapted to our limitations.’*

*(JBL, 72, rural)*

### 3.2. Opportunity

Four themes were identified within the opportunity category, representing external or environmental factors that shape older adults’ ability to participate in social prescribing activities. These include accessibility and transportation, availability of activities in familiar and accessible locations, social interaction and community involvement, and outdoor activities and contact with nature.

#### 3.2.1. Sub-Theme 1: Accessibility and Transportation

Transport accessibility was a consistent barrier to participation—particularly among participants living in rural areas or without personal transportation. The absence of reliable, adapted, or affordable transport options was closely linked to social isolation and disengagement from community activities. Participants described how current public transport services were often infrequent, inaccessible, or mismatched with activity schedules.

Barrier: The lack of available transport was identified as a significant difficulty. Participants said that public transport was not a viable alternative, either because of the lack of options or because the timetables were not suited to their needs.

*‘Transport is a problem. I don’t have a car, and the bus here isn’t an option. When there is one, it’s early in the morning and late in the evening—I can’t be out all day, I have things to do at home.’*

*(MP, 67, rural)*

Facilitator: Several participants suggested that organised and adapted transport, if made available, would significantly facilitate access and encourage participation, especially among those with mobility problems.

*‘If there was transport to take us there and back, that would help a lot. We wouldn’t have to depend on anyone.’*

*(BM, 69, rural)*

#### 3.2.2. Sub-Theme 2: Availability of Activities in Accessible Locations

The location and familiarity of activity settings played a significant role in participants’ willingness to engage. Venues such as parish councils, local halls, and community centres—already embedded in participants’ routines—were seen as safe, comfortable, and trustworthy environments. Participants emphasised the need for even distribution of activities across urban and rural zones, along with clear guidance on where, when, and how to participate.

Barrier: The concentration of activities in the urban centre was mentioned, leaving parishes and towns further away with few options. Participants mentioned that this disparity limits the participation of older people who live outside the centre.

*‘The activities are almost all in the city. There’s hardly anything in the parishes—I don’t even know of any. For those of us farther out, it’s hard to join.’*

*(MLP, 71, urban periphery)*

Facilitator: Holding activities in familiar locations increases comfort and participation among older adults. Choosing spaces that are part of the community’s daily routine fosters a greater sense of safety and belonging. Additionally, providing transport to these locations and clear information on how to access the activities can further enhance engagement.

*‘It’s important that activities are close by, in places we already know—like the parish or the town hall. And if there was transport, that would help a lot.’*

*(JL, 76, rural)*

#### 3.2.3. Sub-Theme 3: Social Interaction and Community Involvement

Opportunities for social connection and community engagement were repeatedly emphasised as essential to emotional and mental well-being. Group activities were seen as protective against loneliness, fostering a sense of belonging and shared identity. Participants valued spaces for peer support, where they could share experiences, form friendships, and re-establish social networks disrupted by ageing or isolation.

However, some also expressed hesitancy or anxiety about entering unfamiliar social spaces, highlighting the importance of creating supportive, familiar, and welcoming environments.

Barrier: Anxiety and fear of new environments were mentioned as factors that hinder adherence. Some participants mentioned that they prefer to stay in more familiar social contexts, as they feel insecure about taking part in new activities.

*‘I don’t feel very motivated—it makes me anxious. I prefer staying at home and chatting with neighbours I already know.’*

*(IM, 97, rural)*

Facilitator: Forming groups and carrying out community activities have been mentioned as effective strategies for strengthening the elderly’s sense of belonging and promoting opportunities for socialisation and mutual support. The involvement of health professionals can facilitate the structuring of these groups and encourage participation.

*‘It would be helpful if you made groups with people from where we live—people we already know. Maybe someone from the parish could help organise it.’*

*(FM, 81, rural)*

#### 3.2.4. Sub-Theme 4: Outdoor Activities and Contact with Nature

Outdoor activities and contact with nature were viewed as powerful contributors to both physical and mental well-being. Participants described activities like walking, gardening, or excursions as uplifting and relaxing, offering both social interaction and therapeutic contact with natural environments. To support inclusive participation, activities must be physically accessible, adapted, and supported when necessary.

Barrier: Physical limitations were mentioned as one of the main obstacles to taking part in outdoor activities. Participants mentioned that mobility difficulties and muscle pain make some activities, such as prolonged walks, challenging and that more accessible alternatives need to be developed.

*‘I’d love to take part, but I can’t walk like I used to. My legs hurt too much—I need something more adapted to my condition.’*

*(JB, 94, urban)*

Facilitator: Activities such as walking, gardening, and outings were highlighted as contributing to the physical and mental well-being of older adults, creating opportunities for contact with nature and socialisation in a pleasant environment.

*‘I wish there were more outdoor activities—In nature. I’ve always worked in the fields. I don’t like all these computer things.’*

*(MCC, 73, rural)*

### 3.3. Motivation

Three themes emerged within the motivation category, reflecting the internal drivers that influence older adults’ engagement in social prescribing. These included the promotion of mental health and emotional well-being, personalisation and diversity of activities, and the perceived support and recognition of older adults as valued participants.

#### 3.3.1. Sub-Theme 1: Promotion of Mental Health and Emotional Well-Being

Mental health and emotional well-being were cited as key motivators for participating in community-based activities. Participants described a desire for structured opportunities that could help combat loneliness, reduce anxiety, and foster personal growth. Activities such as support groups, self-reflection workshops, and creative expression were seen as particularly beneficial for enhancing resilience and emotional balance. Some participants noted that existing programmes were not always designed with older adults in mind.

Barrier: The perception that activities are aimed at a younger audience was identified as a factor that discourages older people from taking part. Some participants said that the programmes available don’t always take into account their needs and expectations, which creates a feeling of exclusion and reduces adherence to the activities.

*‘I often feel like the activities are more for younger people—or people who can move better than I can.’*

*(FN, 82, urban)*

Facilitator: The creation of support groups and spaces dedicated to personal and emotional development can help reduce isolation and strengthen the well-being of older people. Activities such as self-knowledge sessions and artistic expression (e.g., painting, theatre) were mentioned as effective means of sharing experiences and strengthening coping strategies.

*‘It’s important to have activities that support mental health—that’s something I really need. After the pandemic, things got even harder…’*

*(JPP, 75, urban)*

#### 3.3.2. Sub-Theme 2: Personalisation and Diversity of Activities

The personalisation and diversity of available activities significantly influenced motivation. Participants valued options that combined learning, creativity, and meaningful social interaction. Suggestions included activities in arts, music, gardening, and cultural engagement, reflecting the broad range of preferences among older adults.

When activities were perceived as too generic or repetitive, motivation to participate declined. Participants highlighted the need for variety and flexibility to sustain long-term involvement.

Barrier: The lack of adaptation of activities to individual preferences was identified as a demotivating factor. Participants said that many of the options on offer are generalised and do not take into account the different interests and abilities of the elderly.

*‘Sometimes the activities are all the same—they don’t really consider what we enjoy or are capable of.’*

*(IR, 67, urban)*

Facilitator: Offering diversified activities tailored to the individual interests of the elderly promotes greater involvement and satisfaction. Creative and cultural activities were mentioned as effective ways of stimulating participation.

*‘I’d like to see more activities in the arts—like painting or pottery.’*

*(NM, 71, urban)*

#### 3.3.3. Sub-Theme 3: Support and Recognition of Older Adults

Feeling recognised, supported, and heard was a central motivational factor for participants. Many expressed the need for structured feedback mechanisms, where they could contribute ideas and feel that their perspectives were taken seriously. This sense of involvement fostered ownership, trust, and sustained participation.

Barrier: The lack of ongoing support was identified as an obstacle to participation by the elderly. Some said they needed support to stay involved, especially those with mobility difficulties or who felt unsafe travelling alone.

*‘If someone could come with us the first few times, I’d feel more at ease. Going alone, I’m sometimes afraid.’*

*(JJL, 76, rural)*

Facilitator: Holding periodic feedback sessions was mentioned as a strategy that allows activities to be adjusted to the needs of the elderly while at the same time providing them with a space to express opinions and suggestions. This process contributes to the continuous improvement of initiatives and strengthens the involvement of participants.

*‘I’d like meetings like this—where we can speak and be heard—to happen more often.’*

*(FN, 82, urban)*

## 4. Discussion

This study explored the barriers and facilitators influencing older adults’ engagement in social prescribing, using the COM-B model (Capability, Opportunity, Motivation) as a guiding framework [14]. Findings revealed that while numerous challenges persist, strategic attention to key facilitators can significantly enhance participation and adherence among older adults. Social prescribing can be perceived as beneficial to patient well-being, supports healthy ageing, strengthens community cohesion, and contributes to caregiver satisfaction and professional effectiveness [30].

The data suggest that both structural and individual barriers play a decisive role in participation. Key obstacles included lack of accessible transport [2], the concentration of services in urban areas [31] and personal-level challenges such as social anxiety and low digital literacy [12]. These findings are consistent with the broader literature indicating that older adults’ engagement in community-based interventions is shaped by physical, social, emotional, and environmental factors [15,32,33].

Similar results have emerged in international contexts. For example, Sandhu et al. [10] identified that successful liaison officer programmes in the UK depend not only on personalisation but also on systemic supports such as transport and digital inclusion. In East Asia, the studies by Lee et al. and Menhas et al. [9,31] highlight how cultural values and social roles influence the adoption of non-clinical health interventions. These results suggest that many barriers are globally relevant, although influenced by the local context.

In the Portuguese context, these conclusions align with structural challenges already recognised on national political agendas. The lack of decentralisation in service provision and insufficient support for local transport options are particularly critical. Existing initiatives, such as municipal health strategies or ageing plans, could integrate social prescription components, ensuring equitable access to programmes in both rural and urban contexts [21,34].

### 4.1. Capability

Within the capability domain, participants described several internal challenges, particularly physical and health limitations, which restricted access to more physically demanding activities [3]. These findings underscore the importance of adapting activities to varying functional levels, as supported by prior studies advocating for more inclusive, age-friendly programme design [35,36].

Digital exclusion was also a major barrier. Although some older adults recognised the value of technology for connection and learning, many reported low digital literacy and discomfort with devices—a finding echoed by several authors [12,37]. Ongoing digital training and support were cited as essential to enabling full participation [2,38]. This reinforces the need for integrated digital literacy programmes as part of public health strategies aimed at older populations.

Additionally, limited access to clear, tailored information was a recurrent theme. Participants often lacked awareness of available services or how to access them, which hindered engagement. These findings align with previous research identifying lack of accessible information as a major barrier to participation [18]. This gap highlights the need for targeted communication strategies that are culturally and cognitively appropriate for older adults, leveraging familiar community-based platforms such as health units, pharmacies, and local centres [39]. To solve this problem, communication strategies should be adapted to the literacy levels and preferences of the elderly, using reliable local sources such as community pharmacies, health centres, and parish councils [4].

### 4.2. Opportunity

Within the opportunity domain, external and structural conditions—such as geography, mobility, and availability of local services—significantly shaped older adults’ ability to engage. A major theme was the lack of accessible transport, especially among those living in rural or peripheral areas. Participants described infrequent, unreliable public transport and poor scheduling, which severely limited their ability to attend activities [11,40]. This aligns with earlier work identifying transport access as a critical determinant of community engagement and a barrier to participation in social prescribing programmes [2]. From the point of view of public policy, these results underline the urgency of creating transport systems adapted to the needs of the elderly, particularly in municipalities with few resources. Strengthening support at the local level and decentralising services would allow older people to be better integrated into health promotion activities. The implementation of mobile services or interparochial collaboration could improve accessibility.

Another persistent challenge was the urban concentration of activities, leaving rural areas with few opportunities. This geographical imbalance restricts access to social and leisure resources for older adults outside city centres [7,19]. These findings reinforce calls for more equitable, decentralised programming to ensure broader inclusion [31,41]. This decentralisation could be guided by municipal health plans, guaranteeing access based on proximity and personalised programming.

In addition to physical access, social interaction and community involvement were identified as key enablers of participation. Participants emphasised the value of support groups, community-based spaces, and peer networks, which fostered a sense of inclusion and emotional safety [12]. However, for some, the idea of entering unfamiliar environments provoked anxiety or reluctance—especially among those living alone or with limited mobility [13]. These barriers can be addressed by health professionals through gradual exposure strategies, initial follow-up and group input sessions.

These findings underscore the importance of familiar and socially welcoming environments, which have been shown to enhance adherence to social prescribing programmes. Community-based activities—such as arts, sports, personal development, and gardening—encourage adherence and continuity among older adults, especially when facilitated by primary health care professionals, promoting well-being and healthy ageing [42]. As recommended by [43], the use of progressive, confidence-building strategies—such as accompaniment or group introductions—can help ease this transition and promote sustained engagement.

### 4.3. Motivation

The motivation component was driven largely by a desire for mental well-being, personal relevance, and recognition. Participants highlighted the importance of activities that supported emotional health, particularly after prolonged periods of isolation [3,10]. However, some expressed concern that existing activities were not tailored to older populations—an issue that may reinforce disengagement.

A significant barrier mentioned was the lack of personalisation of activities, with many older people saying that the options available did not reflect their individual interests and abilities [8]. These findings are in line with research indicating that offering diversified activities adapted to individual needs increases adherence and satisfaction among the elderly [6,38]. Ensuring user-centred design in social prescription programmes can promote stronger engagement and better emotional outcomes.

Ongoing support and initial accompaniment also emerged as key motivational factors. Some participants noted that the absence of early-stage guidance or emotional reassurance discouraged participation—particularly for those experiencing anxiety or mobility challenges. These concerns are supported by findings from several authors [11,44,45], who suggest that the presence of health professionals or trained volunteers at early sessions can improve confidence and reduce dropout rates. As [12] observe, structured introductory support can facilitate progressive and sustained involvement in social prescribing initiatives. These findings have practical implications for health professionals, especially nurses and community health workers. Nurses can play a key role in identifying eligible older people, offering initial support and ensuring follow-up. Training in motivational interviewing, digital orientation and the coordination of local resources should be included in ongoing training [3,4].

Ultimately, making social prescribing a sustainable and inclusive practice requires not only the design of evidence-based programmes, but also a coordinated investment in professional capacity and political commitment to equity in ageing. Applying these insights can help ensure that community interventions reach those who need them most—especially older people at risk of isolation, digital exclusion or loss of autonomy.

### 4.4. Study Limitations

This study has several limitations that should be considered when interpreting the findings. First, the sample was drawn from a single region in Portugal (Alentejo), which may limit the transferability of results to older adults in different cultural or geographic contexts.

Second, the use of focus groups—while effective for exploring collective perceptions—may have influenced the dynamics of participation. Some individuals may have felt less comfortable sharing personal or dissenting views in a group setting.

In addition, the focus group script was not independently pre-tested with the target population prior to data collection. Although the first focus group served as a pilot and did not require adjustments, the absence of a specific cognitive pre-test may have limited the refinement of the wording or structure of the questions. In addition, the number of questions included in the guide may have been ambitious given the 50 min duration and the size of the group. Although the sessions were structured to encourage interaction, the format may have limited deeper group dynamics, and many of the quotes may reflect individual perspectives rather than group-level consensus.

Third, the study did not include a longitudinal component, making it difficult to assess how barriers and facilitators influence engagement over time or after initial participation.

Finally, the pre-existing relationship between the principal investigator and participants, while useful for recruitment and trust-building, may have introduced a degree of response bias or influenced disclosure.

Future research should consider longitudinal and multi-site qualitative designs to explore how engagement with social prescribing evolves and to better account for contextual variation. Including individual interviews alongside group discussions may also enrich the depth and diversity of perspectives captured.

## 5. Conclusions

This study demonstrates that older adults’ engagement in social prescribing is shaped by a combination of capability, opportunity, and motivation-related factors. Key barriers included physical limitations, digital exclusion, transportation challenges, and lack of personalised activities, while facilitators encompassed tailored programme design, digital support, accessible venues, and strong community ties.

These findings underscore the need for integrated, person-centred strategies that enhance accessibility, foster ongoing support, and recognise the emotional and social dimensions of participation. Social prescribing, when designed inclusively, has the potential to promote health, autonomy, and well-being among older populations—especially those in under-resourced or rural areas.

The study provides practical and policy-relevant insights for designing social prescribing interventions that are responsive to the lived realities of older adults, reinforcing its value as a tool for age-inclusive, holistic healthcare delivery.

## Figures and Tables

**Table 1:** Sociodemographic characteristics of focus group participants.

Focus Group 11			
Name	Age	Gender	Place of Residence
IM	97	Male	Rural
FN	82	Female	Urban
ED	76	Male	Urban
MCC	73	Female	Rural
NM	71	Female	Urban
BM	69	Female	Rural
MCP	68	Female	Urban
JL	76	Male	Rural
**Focus Group 21**			
Name	Age	Gender	Place of Residence
FM	81	Male	Rural
JP	68	Male	Urban
MLP	71	Female	Urban
JB	94	Male	Urban
JBL	72	Male	Rural
MP	67	Female	Rural
JPP	75	Male	Urban
IR	67	Female	Urban

**Table 2:** Focus group semi-structured question guide key questions.

Type of Question	Question
Opening	1. What is your age and where do you live?
	2. In your opinion, what could help or motivate you to participate in social prescribing activities recommended by healthcare professionals? 3. What characteristics of the activities (e.g., schedules, location, type) make it easier to participate? 4. Is there anything healthcare professionals can do to facilitate your participation? 5. What difficulties or obstacles do you think you might face when trying to participate in social prescribing activities? 6. Do you feel that the activities currently available are suited to your needs or interests? 7. What kind of support would be most helpful to encourage participation (e.g., transport, technical support, accompaniment)? 8. What specific activities would you like to see available?
Closing	9. Do you have any comments, suggestions, or observations you’d like to share?

## Data Availability

The data supporting this study’s findings are available from the corresponding author upon reasonable request and in compliance with ethical and confidentiality standards.
